# Comprehensive analysis on the expression profile and prognostic values of Synaptotagmins (SYTs) family members and their methylation levels in gastric cancer

**DOI:** 10.1080/21655979.2021.1951059

**Published:** 2021-07-07

**Authors:** Mei-feng Yang, Xing-Xing Long, Hong-sai Hu, Yu-ling Bin, Xuan-ming Chen, Ben-hua Wu, Quan-zhou Peng, Li-sheng Wang, Jun Yao, De-feng Li

**Affiliations:** a Department of Hematology, Yantian District People’s Hospital, Shenzhen, Guangdong, China; b Department of Hematology, The First Affiliated Hospital of South China of University, South China of University, Hengyang, Hunan, China; c Department of Gastroenterology, The Affiliated Zhuzhou Hospital of Xiangya Medical College, Zhuzhou, Hunan, China; d Department of Gastroenterology, The First Affiliated Hospital of South China of University, South China of University, Hengyang, Hunan, China; e Department of Gastroenterology, Shenzhen People’s Hospital (The Second Clinical Medical College, Jinan University; The First Affiliated Hospital, Southern University of Science and Technology), Shenzhen, Guangdong, China; f Department of Pathology, Shenzhen People’s Hospital (The Second Clinical Medical College, Jinan University; The First Affiliated Hospital, Southern University of Science and Technology), Shenzhen, Guangdong, China

**Keywords:** Synaptotagmin-4, synaptotagmin-9, Synaptotagmin-14, prognostic values, gastric cancer

## Abstract

Synaptotagmins (SYTs), constitute a family of 17 membrane-trafficking protein, palying crucial roles in the development and progression of human cancers. However, only very few studies have investigated the expression profile and prognostic values of SYTs family members in gastric cancer (GC). Therefore, we comprehensively evaluated the expression, methylation, prognosis and immune significance of SYTs family members through bioinformatics analysis from the online databases in GC. The expressions of SYT4, SYT9, and SYT14 were up-regulated, and negatively associated with their methylation levels in GC. Both the over-expression of SYT4, SYT9 and SYT14 and their hypomethylation levels contributed to an unsatisfactory overall survival (OS) and progression-free survival (PFS) in GC. Moreover, the low expressions of several methylation cg sites (cg02795029, cg07581146, cg15149095, cg19922137, cg25371503, cg26158959, cg02269161, cg03226737, cg08185661, cg16437728, cg22723056 and cg24678137) were significantly correlated with an unfavorable OS and PFS in GC. Furthermore, the expression of SYT4, SYT9 and SYT14 played a pivotal role in immune cells infiltration in GC. Collectively, our current finding suggested that SYT4, SYT9 and SYT14 might be potent prognostic indictors and promising immunotherapeutic targets for GC patients.

## Graphical abstract

**Figure:**
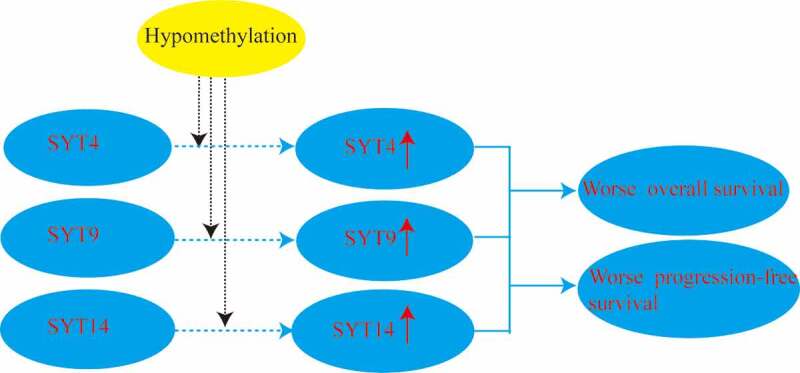

## Introduction

Gastric cancer (GC), worldwide, remains a major threat to human health, with over a million new cases per year [1]. According to the data of the World Health Organization (WHO) in 2018, GC is the fifth most frequently diagnosed cancer, while it is the third cause of cancer-related death [1]. GC usually has symptoms at an advanced stage, which contributes to poor prognosis with a 5-years survival rate of less than 30%[2]. Despite recent progress and advancement in the management of GC, the long-term efficacy is still unfavorable [3]. This is mainly attributed to the unknown pathogenesis of GC [4]. Therefore, it is urgently necessary to identify an effective biomarker for the diagnosis, individualized treatment, and prognosis evaluation of GC.

Synaptotagmins (SYTs) are abundant, evolutionarily conserved integral membrane proteins and constitute a family of 17 isoforms (SYT1～SYT17), which mainly serve as sensors of calcium signaling in cellular secretions [5,6]. The SYT family members are primarily found in tissues and have diverse functional significance [7]. Previous studies have shown that SYT family members play an essential role in postsynaptic receptor endocytosis, vesicle trafficking, membrane repair, synaptic plasticity, and protection against neurodegeneration in the brain [7,8]. Additionally, several studies have recently reported that SYT family members play an oncogenic role in the pathogenesis and progression of human cancers [9,10]. However, the clinical and prognostic significance of SYT family members in the occurrence and development of GC remains largely unknown.

In the present study, we first explored the differential expression and prognostic values of SYT family members in GC *via* The Cancer Genome Atlas (TCGA). Moreover, we evaluated the correlation between the expression s of SYTs family members and methylation levels of their cg sites, and assessed the prognostic significance of their methylation in GC. Univariate and multivariate regression analyses were performed to explore the correlation between the expressions of SYT family members or their methylation levels and clinical characteristics in GC. Finally, Tumor Immune Estimation Resource (TIMER) dataset was used to assess the potential correlation between the expressions of SYT family members and immune cell infiltration in GC. Furthermore, immunohistochemistry (IHC) was used to verify the expressions of SYT proteins in GC tissues and paired normal tissues. Collectively, our current findings revealed the potential prognostic value and biological functionality of SYT family members, which might be important biomarkers of diagnosis and treatment in GC.

## Methods

### Data source

TCGA database is conducted by the National Cancer Institute and National Human Genome Research Institute and contains gene expression database and corresponding clinical information data. The high-throughput sequencing (HTSeq) fragments per kilobase of transcript per million mapped reads (FPKM) data, methylation450 profile, and clinical data with GC were downloaded from the TCGA database via UCSC Xena (https://xena.ucsc.edu/). Therefore, a total of 375 cases of GC and 32 healthy controls were included in this study.

### The mRNA expressions and methylation levels of SYT family members between GC patients and healthy controls

The mRNA expressions of SYT family members were extracted from the HTSeq of GC using Perl 5.26 software. The differential expressions of SYT family members in GC compared with healthy controls were calculated using the ggpubr package in R 4.0 software. The GSE54129 and GSE79973 microarray datasets were obtained from the Gene Expression Omnibus (GEO). The methylation levels of SYT family members were extracted from the methylation450 of GC using Perl 5.26 software. The methylation levels of cg sites in the DNA promoter regions of SYT family members in GC were analyzed using *plyr* and *ggpubr* packages in R 4.0 software.

### Correlation between the mRNA expressions and methylation levels of SYT family members in CG

The relationship between the expressions of SYT family members at the mRNA level and methylation levels of their cg sites was evaluated through *ggplot2* and *ggpubr* packages in R 4.0 software.

### Survival analysis of the mRNA expressions and methylation levels of cg sites of SYT family members

According to the median values of mRNA expressions and methylation levels of SYT family members, GC patients were divided into high- and low-expression groups. Survival analysis was performed to compare high-expression and low-expression groups using the *survival* and *survminer* package in R 4.0 software.

### TIMER analysis

TIMER is an online tool, which can comprehensively assess the immune infiltration status of different cancer types. Therefore, the TIMER software was utilized to explore the correlation between the expressions of SYT family members and six immune infiltration fluids [B cells, CD4^+^ T cells, CD8^+^ T cells, neutrophils, macrophages, and dendritic cells (DCs)].

### Immunohistochemistry (IHC)

The IHC was performed as previously described [11]. Antibodies against SYT4, SYT9, and SYT14 were purchased from Hangzhou HuaAn Biotechnology Co.,Ltd (Zhejiang, China). Goat anti-rabbit IgG were provided by BioTNT (Shanghai, China). Antibodies dilutions of SYT4, SYT9, and SYT14 were 1:1000, 1:1000 and 1:1000.

### Statistical analysis

All statistical analyses and plots were performed using R 4.0. Based on the between-group differences, categorical variables were calculated using the Chi-squared test or Fisher’s exact test. Continuous variables were calculated using the Student’s *t*-test or Mann-Whitney test. Pearson^’^s correlation analysis was adopted for the relationship analysis between the mRNA expressions of SYT family members and their methylation levels. Kaplan-Meier analysis was used to evaluate prognostic factors. *P* < 0.05 was considered statistically significant.

## Results

### Expression status of SYT family members in GC

To examine the expressions of SYT family members, we examined the expressions of SYT family members at the mRNA level in 375 GC patients and 32 healthy controls, which originated from the TCGA database.

**Figure 1.**
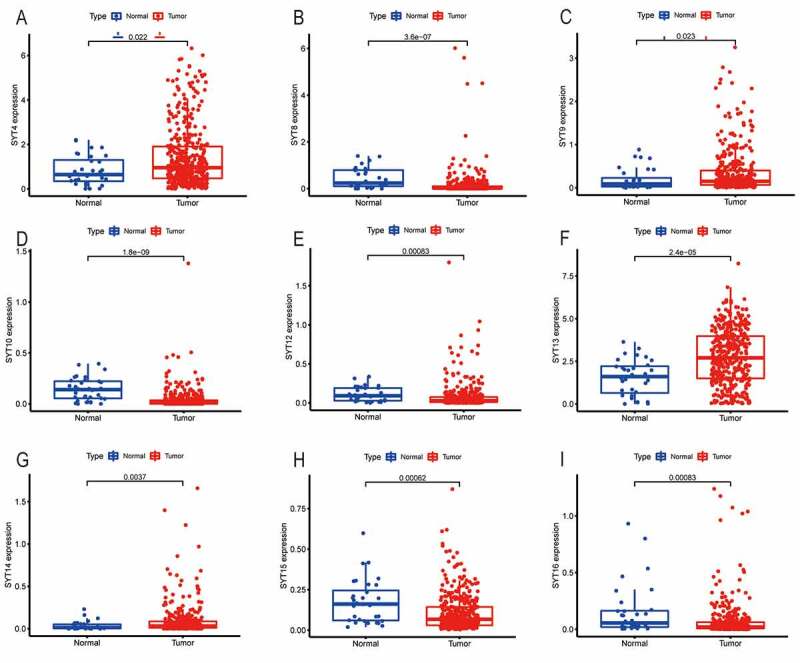
The mRNA expressions of SYT family members in GC patients compared with healthy controls from the TCGA database. SYT4, SYT9, SYT13, and SYT14 were up-regulated in GC (a, c, f, and g). SYT8, SYT10, SYT12, SYT15, and SYT16 were down-regulated in GC (b, d, e, h, and i)

Figure 1 shows that SYT4, SYT9, SYT13, and SYT14 were significantly up-regulated in GC samples compared with the healthy controls (P = 0.022, P = 0.023, P < 0.001, and P = 0.037, respectively), while SYT8, SYT10, SYT12, SYT15, and SYT16 were significantly down-regulated in GC samples (P < 0.001, P < 0.001, P = 0.00083, P = 0.00062, and P = 0.00083, respectively). However, there were no significant differences in the expressions of SYT1, SYT2, SYT3, SYT5, SYT6, SYT7, SYT11, and SYT17 between the GC samples and healthy controls (P = 0.53, P = 0.46, P = 0.92, P = 0.093, P = 0.84, P = 0.43, P = 0.99, and P = 0.28, respectively) (Figure S1).

### Validation of the prognostic values of SYT family members in GC

We utilized Kaplan-Meier survival analysis to evaluate the prognostic values of SYT family members and assess the overall survival (OS) and progression-free survival (PFS) in GC patients from the TCGA database.

**Figure 2.**
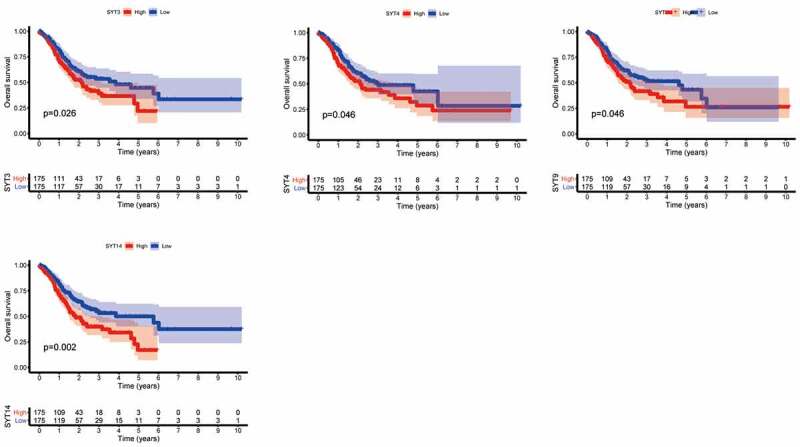
The correlation between the OS and the mRNA expressions of SYT family members in GC patients. The high expressions of SYT3, SYT4, SYT9, and SYT14 were associated with a worse OS (a, b, c, and d)

**Figure 3.**
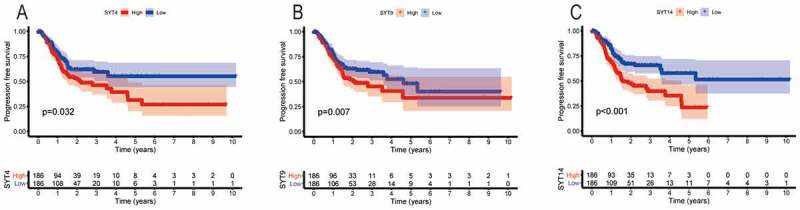
The correlation between the PFS and the mRNA expressions SYT family members in GC patients. The high expressions of SYT4, SYT9, and SYT14 were associated with a worse PFS (a, b, and c)

We found that the high expressions of SYT3, SYT4, SYT9, and SYT14 were associated with a worse OS (P = 0.026, P = 0.046, P = 0.046, and P = 0.002, respectively) (Figure 2), whereas the expressions of SYT1, SYT2, SYT5, SYT6, SYT7, SYT8, SYT10, SYT11, SYT12, SYT13, SYT15, SYT16, and SYT17 were not correlated with OS for GC patients (P = 0.248, P = 0.897, P = 0.585, P = 0.248, P = 0.748, P = 0.164, P = 0.778, P = 0.662, P = 0.115, P = 0.221, P = 0.064, P = 0.414, and P = 0.794, respectively) (Figure S2). Moreover, we found that up-regulated SYT4, SYT9, and SYT14 were significantly correlated with a worse PFS (P = 0.032, P = 0.007, and P < 0.001, respectively) (Figure 3), whereas the expressions of SYT1, SYT2, SYT3, SYT5, SYT6, SYT7, SYT8, SYT10, SYT11, SYT12, SYT13, SYT15, SYT16, and SYT17 were not associated with PFS for GC patients (P = 0.994, P = 0.059, P = 0.124, P = 0.094, P = 0.182, P = 0.611, P = 0.514, P = 0.289, P = 0.141, P = 0.08, P = 0.398, P = 0.069, P = 0.051, and P = 0.064) (Figure S3). Based on the above-mentioned results, we obtained SYT4, SYT9, and SYT14 as candidate genes for further research. Meanwhile, it was shown that the expressions of SYT4, SYT9, and SYT14 were up-regulated in GSE54129 and GSE79973 (Figure S4).

### Correlation between the expressions of SYT family members and methylation levels in GC

DNA methylation of the promoter region is one of the crucial factors that regulate gene expression during the pathogenesis of various cancers. In the present study, we assessed the methylation cg sites of SYT4, SYT9, and SYT14 in GC from the TCGA database.

**Figure 4.**
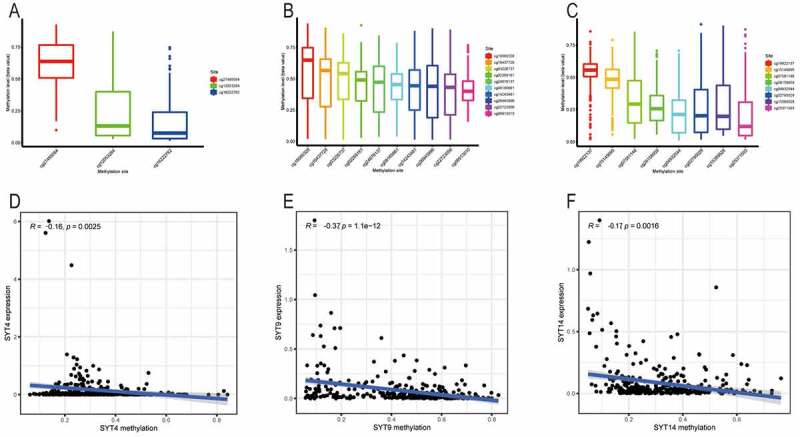
Correlation between the expressions of SYT family members and their methylation levels in GC. A, The distribution of SYT4 DNA promoter CpG sites. B, The distribution of SYT9 DNA promoter CpG sites. C, The distribution of SYT14 DNA promoter CpG sites. D, The expression of SYT4 at the mRNA level was negatively correlated with its methylation level. E, The expression of SYT9 at the mRNA level was negatively correlated with its methylation level. F, The expression of SYT14 at the mRNA level was negatively correlated with its methylation level

There were three (cg27485084, cg12053284, and cg16222762), 10 (cg08913010, (cg02269161, cg03226737, cg08185661, cg14243481, cg16437728, cg18560328, cg22723056, cg24678137, and cg26945996), and eight (cg04932544, cg15389528, cg02795029, cg07581146, cg15149095, cg19922137, cg25371503, and cg26158959) methylation cg sites in SYT4, SYT9, and SYT14, respectively (Figure 4(a), 4(b), and 4(c)). The Pearson^’^s correlation analysis demonstrated that there was an inverse correlation between the mRNA expressions and methylation levels of SYT4, SYT9, and SYT14 (R = −0.16, P = 0,0025; R = −0.37, P < 0.001, and R = −0.17, P = 0.0016, respectively)(Figure 4(d), 4(e), and 4(f)).

**Figure 5.**
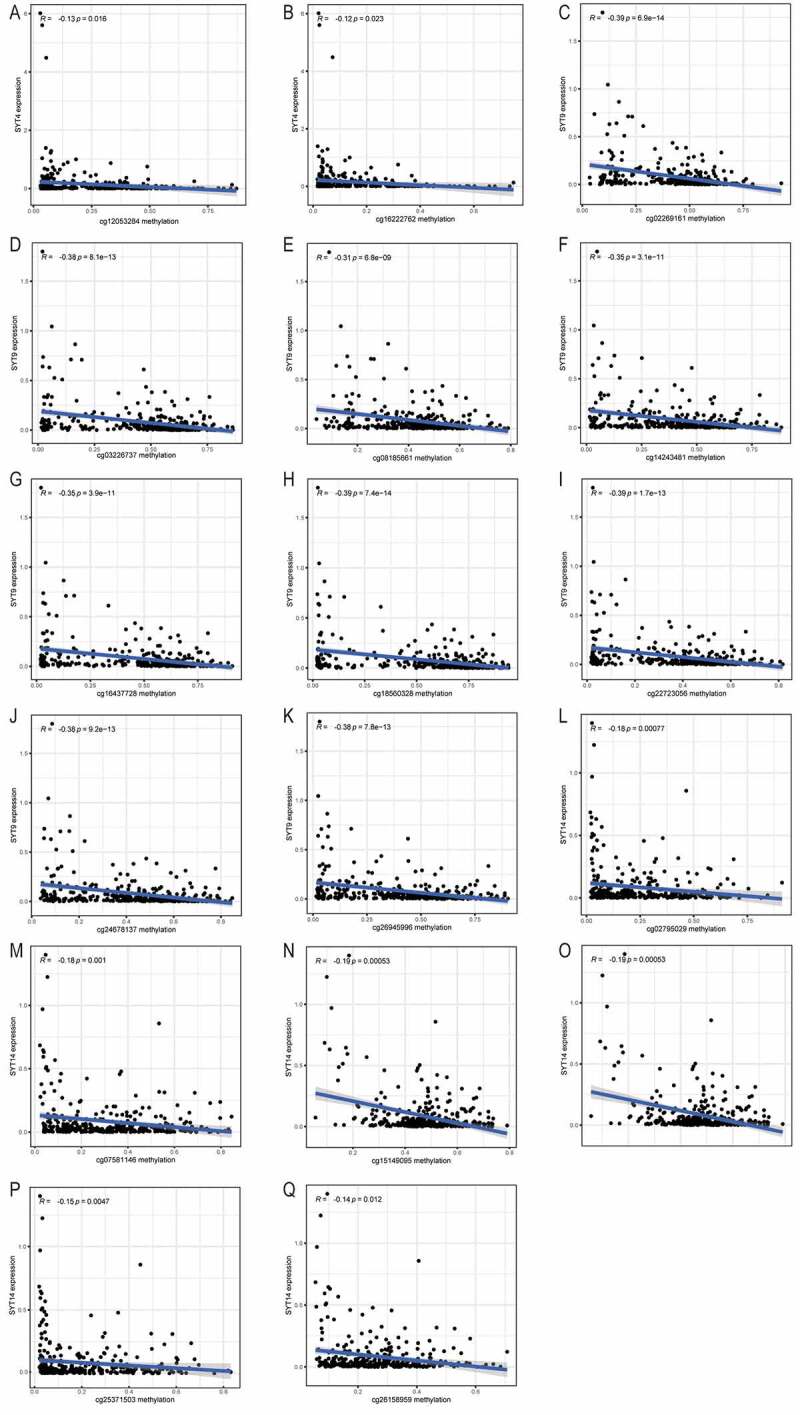
Correlation between the expressions of SYT family members and methylation levels of their cg sites in GC

Indeed, we found that except for one methylation cg site (cg27485084) (P = 0.12) (Figure S5A), the other two methylation cg sites (cg12053284 and cg16222762) were negatively correlated with the expression of SYT4 (P = 0.016 and P = 0.023) (Figure 5(a) and 5(b)). Moreover, we showed that except for one methylation cg site (cg08913010) (P = 0.38) (Figure S5B), the other nine methylation cg sites (cg02269161, cg03226737, cg08185661, cg14243481, cg16437728, cg18560328, cg22723056, cg24678137, and cg26945996) were inversely correlated with the expression of SYT9 (all P < 0.001) (Figure 5(c–j), and 5(k)). Furthermore, we demonstrated that except for two methylation cg sites (cg04932544 and cg15389528) (P = 0.13 and P = 0.051) (Figure S5C and S5D), the other six methylation cg sites (cg02795029, cg07581146, cg15149095, cg19922137, cg25371503, and cg26158959) were negatively associated with the expression of SYT14 (all P < 0.001) (Figure 5(l), 5(m–p), and 5(q)).

### Validation of the prognostic values of different methylation cg sites in GC

Kaplan-Meier survival analysis was used to investigate the prognostic values of methylation cg sites of SYT4, SYT9, and SYT14 in GC patients from the TCGA database.

**Figure 6.**
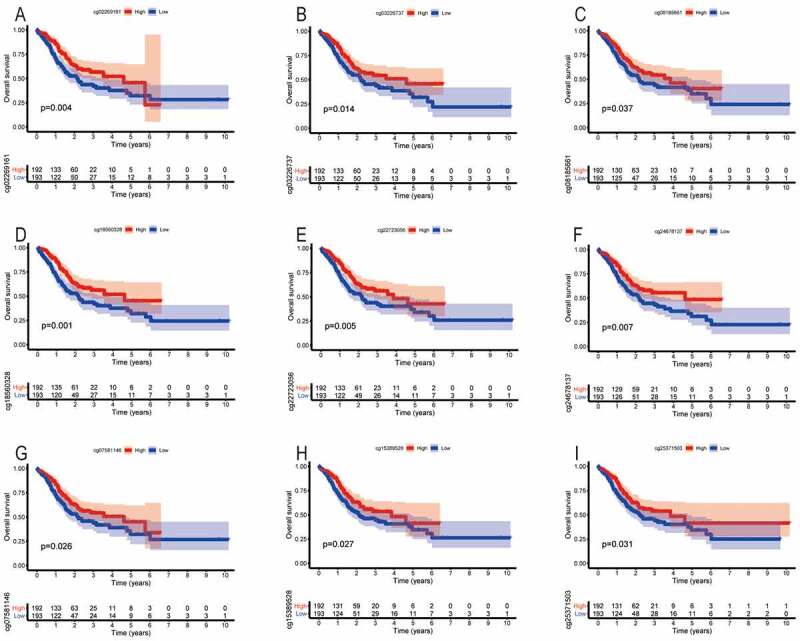
The correlation between the OS and the methylation cg sites of SYT family members in GC patients. The low methylation levels of cg sites (cg02269161, cg03226737, cg08185661, cg18560328, cg22723056, and cg24678137) of SYT9 contributed to an unfavorable OS (a, b, c, d, e, and f). The low methylation levels of cg sites (cg07581146, cg15389528, and cg25371503) of SYT14 contributed to an unsatisfactory OS (G, h, and i)

We found that the methylation levels of cg sites (cg27485084, cg12053284, and cg16222762) of SYT4 were not associated with OS (P = 0.108, P = 0.283, and P = 0.145, respectively) (Figure S6A, S6B, and S6C). Moreover, we showed that the low methylation levels of cg sites (cg02269161, cg03226737, cg08185661, cg18560328, cg22723056, and cg24678137) of SYT9 were correlated with an unfavorable OS (P = 0.004, P = 0.014, P = 0.037, P = 0.001, P = 0.005, and P = 0.007, respectively) (Figure 6(a–e) and 6(f)), whereas other methylation cg sites (cg08913010, cg14243481, cg16437728, and cg26945996) did not have a prognostic value for GC patients (P = 0.926, P = 0.065, P = 0.059, and P = 0.056, respectively) (Figure S6D, S6E, S6F and S6G). Furthermore, we demonstrated that the low methylation levels of cg sites (cg07581146, cg15389528, and cg25371503) of SYT14 were associated with an unsatisfactory OS (P = 0.026, P = 0.027, and P = 0.031, respectively) (Figure 6(g,h), and 6(i)), whereas other methylation cg sites (cg02795029, cg04932544, cg15149095, cg19922137, and cg26158959) were not associated with a prognostic value for GC patients (P = 0.160, P = 0.390, P = 0.382, P = 0.346, and P = 0.164) (Figure S6H, S6I, S6J, S6K, and S6L).

**Figure 7.**
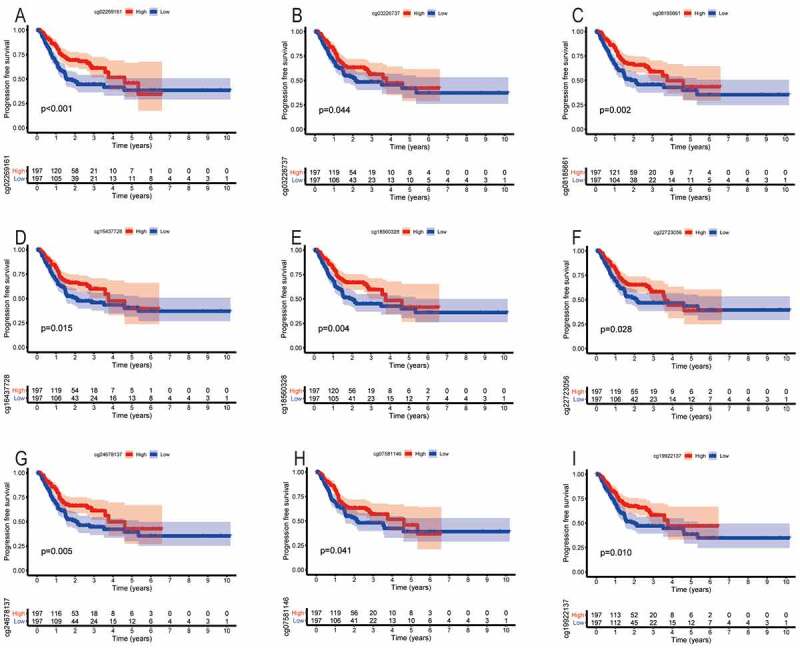
The correlation between the PFS and the methylation cg sites of SYT family members in GC patients. The low methylation levels of cg sites (cg02269161, cg03226737, cg08185661, cg16437728, cg18560328, cg22723056, and cg24678137) of SYT9 were associated with an unfavorable PFS (a, b, c, d, e, f, and g). The low methylation levels of cg sites (cg07581146 and cg19922137) of SYT14 were correlated with an unfavorable PFS (h and i)

Similarly, we found that the methylation levels of cg sites (cg27485084, cg12053284, and cg16222762) of SYT4 were not associated with PFS (P = 0.359, P = 0.828, and P = 0.584, respectively) (Figure S7A, S7B, and S7C). Moreover, we showed that the low methylation levels of cg sites (cg02269161, cg03226737, cg08185661, cg16437728, cg18560328, cg22723056, and cg24678137) of SYT9 were associated with an unfavorable PFS (P < 0.001, P = 0.044, P = 0.002, P = 0.015, P = 0.004, P = 0.028, and P = 0.005, respectively) (Figure 7(a–f) and 7(g)), whereas other methylation cg sites (cg08913010, cg14243481, and cg26945996) were not associated with a prognostic value for GC patients (P = 0.564, P = 0.075, and P = 0.122, respectively) (Figure S7D, S7E, and S7F). Furthermore, we demonstrated that the low methylation levels of cg sites (cg07581146 and cg19922137) of SYT14 were correlated with an unfavorable PFS (P = 0.041 and P = 0.010) (Figure 7(h) and 7(i)), whereas other methylation cg sites (cg02795029, cg04932544, cg15149095, cg15389528, cg25371503, and cg26158959) were not correlated with a prognostic value for GC patients (P = 0.112, P = 0.139, P = 0.635, P = 0.059, P = 0.205, and P = 0.061, respectively) (Figure S7G, S7H, S7I, S7J, S7K, and S7L).

### Association between the mRNA expression or methylation level and clinicopathologic characteristics

We further explored the detailed association between the expressions of SYT4, SYT9, and SYT14, or their methylation levels, and clinicopathologic characteristics.

Table 1 and Table 2 show that the expression of SYT4 was closely correlated with the T stage (P = 0.0029) and pathological stage (P = 0.0181), while the methylation level of SYT4 was closely correlated with the T stage (P = 0.0192). Table 3 and Table 4 reveal that the expression of SYT9 was closely associated with the T stage (P = 0.0159). Meanwhile, the methylation level of SYT9 was closely associated with the M stage (P = 0.0032). Table 5 and Table 6 indicate that the expression of SYT14 was closely correlated with the T stage (P = 0.0129). Besides, the methylation level of SYT14 was closely correlated with age (P = 0.0208) and pathological stage (P = 0.0273).

**Table 1.** Correlation between SYT4 mRNA expression and clinicopathologic characteristics in TCGA database

		Total	High	Low	*P*-value
Age ^a^	≤65	155(45.86%)	84(49.7%)	71(42.01%)	0.156
Age	>65	179(52.96%)	82(48.52%)	97(57.4%)	
Age	unknow	4(1.18%)	3(1.78%)	1(0.59%)	
Gender ^a^	female	118(34.91%)	60(35.5%)	58(34.32%)	0.9092
Gender	male	220(65.09%)	109(64.5%)	111(65.68%)	
Grade ^a^	G2	123(36.39%)	46(27.22%)	77(45.56%)	
Grade	G3	198(58.58%)	114(67.46%)	84(49.7%)	
Grade	unknow	9(2.66%)	5(2.96%)	4(2.37%)	
M ^a^	M0	301(89.05%)	148(87.57%)	153(90.53%)	0.6162
M	M1	19(5.62%)	11(6.51%)	8(4.73%)	
M	unknow	18(5.33%)	10(5.92%)	8(4.73%)	
N ^a^	N0	103(30.47%)	45(26.63%)	58(34.32%)	0.3329
N	N1	87(25.74%)	44(26.04%)	43(25.44%)	
N	N2	71(21.01%)	35(20.71%)	36(21.3%)	
N	N3	69(20.41%)	40(23.67%)	29(17.16%)	
N	unknow	8(2.37%)	5(2.96%)	3(1.78%)	
T ^a^	T1	18(5.33%)	3(1.78%)	15(8.88%)	0.0029
T	T2	67(19.82%)	27(15.98%)	40(23.67%)	
T	T3	160(47.34%)	84(49.7%)	76(44.97%)	
T	T4	93(27.51%)	55(32.54%)	38(22.49%)	
Stage ^a^	Stage I	46(13.61%)	13(7.69%)	33(19.53%)	0.0181
Stage	Stage II	108(31.95%)	58(34.32%)	50(29.59%)	
Stage	Stage III	145(42.9%)	73(43.2%)	72(42.6%)	
Stage	Stage IV	29(8.58%)	17(10.06%)	12(7.1%)	
Stage	unknow	10(2.96%)	8(4.73%)	2(1.18%)	

a, Chi-squared test

**Table 2.** Correlation between SYT4 methylation levels and clinicopathologic characteristics in TCGA database

		Total	High	Low	*P-*value
Age ^a^	≤65	155(45.86%)	70(41.42%)	85(50.3%)	0.1245
Age	>65	179(52.96%)	97(57.4%)	82(48.52%)	
Age	unknow	4(1.18%)	2(1.18%)	2(1.18%)	
Gender ^a^	female	118(34.91%)	51(30.18%)	67(39.64%)	0.087
Gender	male	220(65.09%)	118(69.82%)	102(60.36%)	
Grade ^a^	G1	8(2.37%)	3(1.78%)	5(2.96%)	0.2182
Grade	G2	123(36.39%)	56(33.14%)	67(39.64%)	
Grade	G3	198(58.58%)	108(63.91%)	90(53.25%)	
Grade	unknow	9(2.66%)	2(1.18%)	7(4.14%)	
M ^a^	M0	301(89.05%)	151(89.35%)	150(88.76%)	1
M	M1	19(5.62%)	10(5.92%)	9(5.33%)	
M	unknow	18(5.33%)	8(4.73%)	10(5.92%)	
N ^a^	N0	103(30.47%)	52(30.77%)	51(30.18%)	0.5057
N	N1	87(25.74%)	43(25.44%)	44(26.04%)	
N	N2	71(21.01%)	40(23.67%)	31(18.34%)	
N	N3	69(20.41%)	30(17.75%)	39(23.08%)	
N	unknow	8(2.37%)	4(2.37%)	4(2.37%)	
T ^a^	T1	18(5.33%)	11(6.51%)	7(4.14%)	0.0192
T	T2	67(19.82%)	28(16.57%)	39(23.08%)	
T	T3	160(47.34%)	77(45.56%)	83(49.11%)	
T	T4	93(27.51%)	53(31.36%)	40(23.67%)	
Stage ^a^	Stage I	46(13.61%)	18(10.65%)	28(16.57%)	0.2368
Stage	Stage II	108(31.95%)	55(32.54%)	53(31.36%)	
Stage	Stage III	145(42.9%)	79(46.75%)	66(39.05%)	
Stage	Stage IV	29(8.58%)	12(7.1%)	17(10.06%)	
Stage	unknow	10(2.96%)	5(2.96%)	5(2.96%)	

a, Chi-squared test

**Table 3.** Correlation between SYT9 mRNA expression and clinicopathologic characteristics in TCGA database

		Total	High	Low	*P*-value
Age ^a^	≤65	155(45.86%)	88(52.07%)	67(39.64%)	0.065
Age	>65	179(52.96%)	81(47.93%)	98(57.99%)	
Age	unknow	4(1.18%)	0(0%)	4(2.37%)	
Gender ^a^	female	118(34.91%)	53(31.36%)	65(38.46%)	0.2094
Gender	male	220(65.09%)	116(68.64%)	104(61.54%)	
Grade ^a^	G2	123(36.39%)	48(28.4%)	75(44.38%)	
Grade	G3	198(58.58%)	113(66.86%)	85(50.3%)	
Grade	unknow	9(2.66%)	4(2.37%)	5(2.96%)	
M ^a^	M0	301(89.05%)	144(85.21%)	157(92.9%)	0.2897
M	M1	19(5.62%)	12(7.1%)	7(4.14%)	
M	unknow	18(5.33%)	13(7.69%)	5(2.96%)	
N ^a^	N0	103(30.47%)	52(30.77%)	51(30.18%)	0.9095
N	N1	87(25.74%)	41(24.26%)	46(27.22%)	
N	N2	71(21.01%)	37(21.89%)	34(20.12%)	
N	N3	69(20.41%)	36(21.3%)	33(19.53%)	
N	unknow	8(2.37%)	3(1.78%)	5(2.96%)	
T ^a^	T1	18(5.33%)	6(3.55%)	12(7.1%)	0.0159
T	T2	67(19.82%)	30(17.75%)	37(21.89%)	
T	T3	160(47.34%)	79(46.75%)	81(47.93%)	
T	T4	93(27.51%)	54(31.95%)	39(23.08%)	
Stage ^a^	Stage I	46(13.61%)	21(12.43%)	25(14.79%)	0.5991
Stage	Stage II	108(31.95%)	49(28.99%)	59(34.91%)	
Stage	Stage III	145(42.9%)	76(44.97%)	69(40.83%)	
Stage	Stage IV	29(8.58%)	16(9.47%)	13(7.69%)	
Stage	unknow	10(2.96%)	7(4.14%)	3(1.78%)	

a, Chi-squared test

**Table 4.** Correlation between SYT9 methylation levels and clinicopathologic characteristics in TCGA database

		Total	High	Low	*P*-value
Age ^a^	≤65	155(45.86%)	66(39.05%)	89(52.66%)	0.0208
Age	>65	179(52.96%)	100(59.17%)	79(46.75%)	
Age	unknow	4(1.18%)	3(1.78%)	1(0.59%)	
Gender ^a^	female	118(34.91%)	59(34.91%)	59(34.91%)	1
Gender	male	220(65.09%)	110(65.09%)	110(65.09%)	
Grade ^a^	G1	8(2.37%)	5(2.96%)	3(1.78%)	0.5796
Grade	G2	123(36.39%)	66(39.05%)	57(33.73%)	
Grade	G3	198(58.58%)	97(57.4%)	101(59.76%)	
Grade	unknow	9(2.66%)	1(0.59%)	8(4.73%)	
M ^a^	M0	301(89.05%)	161(95.27%)	140(82.84%)	0.0032
M	M1	19(5.62%)	3(1.78%)	16(9.47%)	
M	unknow	18(5.33%)	5(2.96%)	13(7.69%)	
N ^a^	N0	103(30.47%)	60(35.5%)	43(25.44%)	0.2003
N	N1	87(25.74%)	38(22.49%)	49(28.99%)	
N	N2	71(21.01%)	37(21.89%)	34(20.12%)	
N	N3	69(20.41%)	32(18.93%)	37(21.89%)	
N	unknow	8(2.37%)	2(1.18%)	6(3.55%)	
T ^a^	T1	18(5.33%)	11(6.51%)	7(4.14%)	0.3756
T	T2	67(19.82%)	28(16.57%)	39(23.08%)	
T	T3	160(47.34%)	84(49.7%)	76(44.97%)	
T	T4	93(27.51%)	46(27.22%)	47(27.81%)	
Stage ^a^	Stage I	46(13.61%)	22(13.02%)	24(14.2%)	0.1083
Stage	Stage II	108(31.95%)	61(36.09%)	47(27.81%)	
Stage	Stage III	145(42.9%)	73(43.2%)	72(42.6%)	
Stage	Stage IV	29(8.58%)	9(5.33%)	20(11.83%)	
Stage	unknow	10(2.96%)	4(2.37%)	6(3.55%)	

a, Chi-squared test

**Table 5.** Correlation between SYT14 mRNA expression and clinicopathologic characteristics in TCGA database

		Total	High	Low	*P*-value
Age ^a^	≤65	155(45.86%)	81(47.93%)	74(43.79%)	0.6493
Age	>65	179(52.96%)	88(52.07%)	91(53.85%)	
Age	unknow	4(1.18%)	0(0%)	4(2.37%)	
Gender ^a^	female	118(34.91%)	56(33.14%)	62(36.69%)	0.5683
Gender	male	220(65.09%)	113(66.86%)	107(63.31%)	
Grade ^a^	G1	8(2.37%)	4(2.37%)	4(2.37%)	0.4257
Grade	G2	123(36.39%)	56(33.14%)	67(39.64%)	
Grade	G3	198(58.58%)	105(62.13%)	93(55.03%)	
Grade	unknow	9(2.66%)	4(2.37%)	5(2.96%)	
M ^a^	M0	301(89.05%)	151(89.35%)	150(88.76%)	1
M	M1	19(5.62%)	9(5.33%)	10(5.92%)	
M	unknow	18(5.33%)	9(5.33%)	9(5.33%)	
N ^a^	N0	103(30.47%)	52(30.77%)	51(30.18%)	0.9068
N	N1	87(25.74%)	46(27.22%)	41(24.26%)	
N	N2	71(21.01%)	34(20.12%)	37(21.89%)	
N	N3	69(20.41%)	33(19.53%)	36(21.3%)	
N	unknow	8(2.37%)	4(2.37%)	4(2.37%)	
T ^a^	T1	18(5.33%)	6(3.55%)	12(7.1%)	0.0129
T	T2	67(19.82%)	26(15.38%)	41(24.26%)	
T	T3	160(47.34%)	94(55.62%)	66(39.05%)	
T	T4	93(27.51%)	43(25.44%)	50(29.59%)	
Stage ^a^	Stage I	46(13.61%)	18(10.65%)	28(16.57%)	0.2135
Stage	Stage II	108(31.95%)	57(33.73%)	51(30.18%)	
Stage	Stage III	145(42.9%)	76(44.97%)	69(40.83%)	
Stage	Stage IV	29(8.58%)	11(6.51%)	18(10.65%)	
Stage	unknow	10(2.96%)	7(4.14%)	3(1.78%)	

a, Chi-squared test

**Table 6.** Correlation between SYT14 methylation levels and clinicopathologic characteristics in TCGA database

		Total	High	Low	*P*-value
Age ^a^	≤65	155(45.86%)	66(39.05%)	89(52.66%)	0.0208
Age	>65	179(52.96%)	100(59.17%)	79(46.75%)	
Age	unknow	4(1.18%)	3(1.78%)	1(0.59%)	
Gender ^a^	female	118(34.91%)	56(33.14%)	62(36.69%)	0.5683
Gender	male	220(65.09%)	113(66.86%)	107(63.31%)	
Grade ^a^	G1	8(2.37%)	2(1.18%)	6(3.55%)	0.3453
Grade	G2	123(36.39%)	63(37.28%)	60(35.5%)	
Grade	G3	198(58.58%)	101(59.76%)	97(57.4%)	
Grade	unknow	9(2.66%)	3(1.78%)	6(3.55%)	
M ^a^	M0	301(89.05%)	157(92.9%)	144(85.21%)	0.0513
M	M1	19(5.62%)	5(2.96%)	14(8.28%)	
M	unknow	18(5.33%)	7(4.14%)	11(6.51%)	
N ^a^	N0	103(30.47%)	60(35.5%)	43(25.44%)	0.0912
N	N1	87(25.74%)	40(23.67%)	47(27.81%)	
N	N2	71(21.01%)	39(23.08%)	32(18.93%)	
N	N3	69(20.41%)	28(16.57%)	41(24.26%)	
N	unknow	8(2.37%)	2(1.18%)	6(3.55%)	
T ^a^	T1	18(5.33%)	11(6.51%)	7(4.14%)	0.5695
T	T2	67(19.82%)	31(18.34%)	36(21.3%)	
T	T3	160(47.34%)	77(45.56%)	83(49.11%)	
T	T4	93(27.51%)	50(29.59%)	43(25.44%)	
Stage ^a^	Stage I	46(13.61%)	23(13.61%)	23(13.61%)	0.0273
Stage	Stage II	108(31.95%)	60(35.5%)	48(28.4%)	
Stage	Stage III	145(42.9%)	74(43.79%)	71(42.01%)	
Stage	Stage IV	29(8.58%)	7(4.14%)	22(13.02%)	
Stage	unknow	10(2.96%)	5(2.96%)	5(2.96%)	

a, Chi-squared test

### Association between the expression of SYT family members and immune cell infiltration

According to increasing evidence on the correlations between immune cell infiltration and prognosis in cancer, we utilized the TIMER database to evaluate the association between the expressions of SYT4, SYT9, and SYT14 and immune cell infiltration.

**Figure 8.**
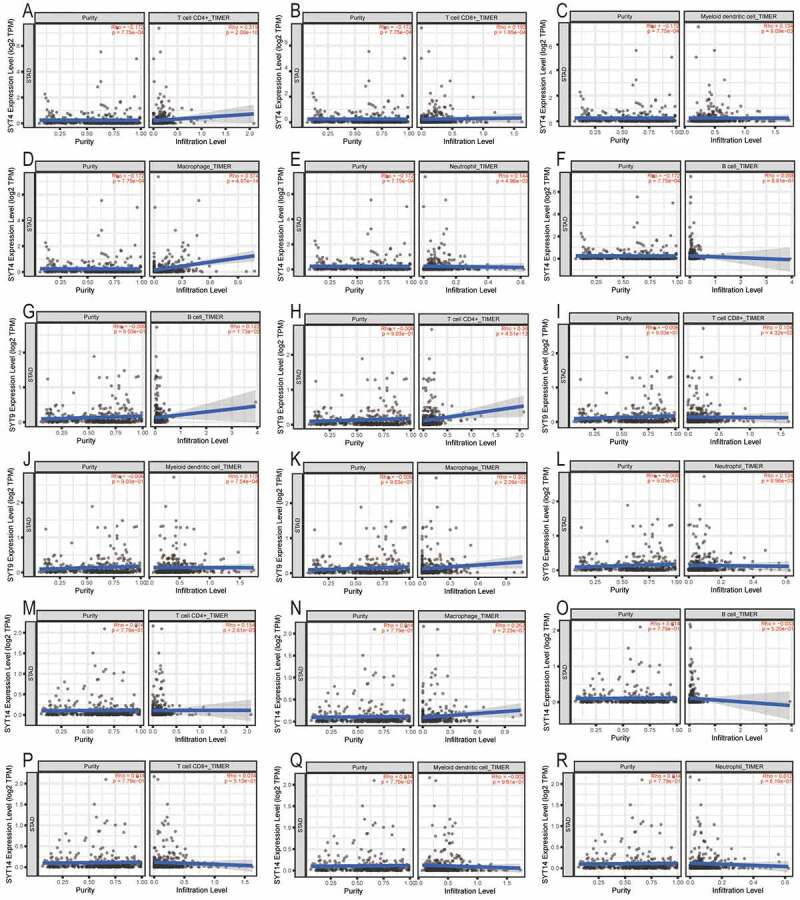
Association between the expressions of SYT family members and tumor infiltration of immune cells (B cells, CD4^+^T cells, CD8^+^T cells, DCs, macrophages, and neutrophils) in GC patients using the TIMER database. Tumor purity is shown in the panels on the left

**Figure 9.**
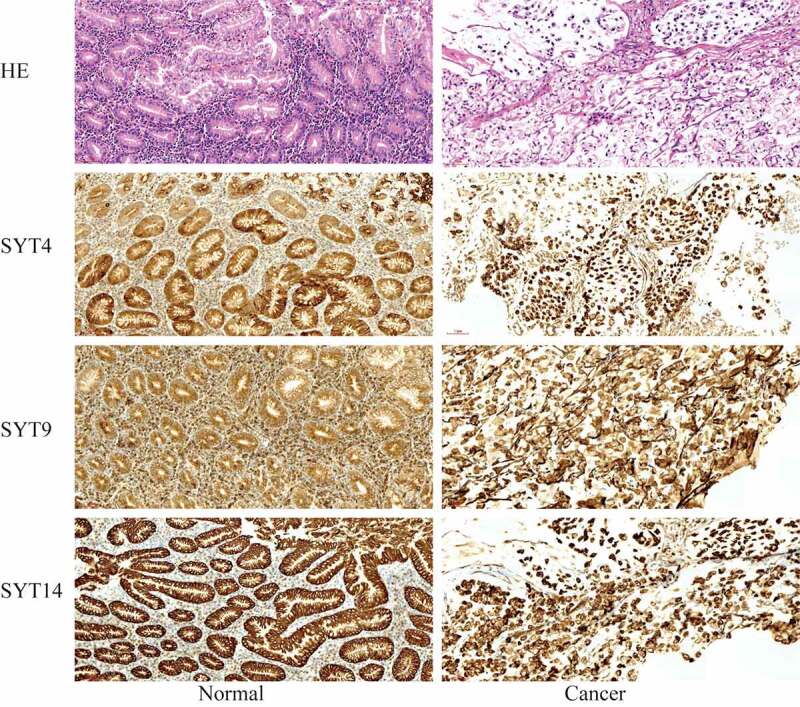
The expressions of SYT4, SYT9, and SYT14 at the protein level. SYT4, SYT9, and SYT14 were over-expressed in GC

Figure 8(a–d), and 8(e) show that the expression of SYT4 was positively correlated with the infiltration of CD4^+^T cells, CD8^+^T cells, DCs, macrophages, and neutrophils (all P < 0.001), whereas it was not correlated with B cells (P = 0.881) (figure 8(f)). Figure 8(g–k), and 8(l) show that the expression of SYT9 was positively associated with the infiltration of B cells, CD4^+^T cells, CD8^+^T cells, DCs, macrophages, and neutrophils (all P < 0.001). Figure 9(m) and 9(n) indicate that the expression of SYT14 was positively associated with the infiltration of CD4^+^T cells and macrophages (all p < 0.001), whereas it was not associated with the infiltration of B cells, CD8^+^ T cells, DCs, and neutrophils (P = 0.52, P = 0.51, P = 0.96, and P = 0.81, respectively) (Figure 8(o–q), and 8(r)).

### SYT4, SYT9, and SYT14 are up-regulated in GC

We examined the expressions of SYT4, SYT9, and SYT14 at the protein level in GC tissues and paired normal gastric tissues using IHC (n = 20). Moreover, we showed that the expressions of SYT4, SYT9, and SYT14 proteins were up-regulated in the GC tissues (Figure 9).

## Discussion

SYT family members are abundant, evolutionarily conserved integral membrane proteins, which play crucial roles in regulated exocytosis in nervous and endocrine systems [8]. However, several studies have recently reported that SYT family members play vital roles in the pathogenesis of human cancers. It has been reported that SYT13 is up-regulated in the lung adenocarcinoma as well as in colorectal cancer, which promotes the proliferation and migration of lung adenocarcinoma cells and colorectal cancer cells and contributes to an unsatisfactory prognosis [10,12]. Sung HY et al. have demonstrated that SYT2 is up-regulated in ovarian cancer and promotes the migration and invasiveness of ovarian carcinoma cells, and is associated with poor survival for patients with ovarian cancer [13]. SYT7 is overexpressed in lung cancer as well as in colorectal cancer and glioma, which promotes cell proliferation, inhibits cell apoptosis, and results in unfavorable prognosis [14–16]. SYT4 is up-regulated in triple-negative breast cancer, confers paclitaxel resistance, and leads to poor prognosis [17]. However, very few studies have evaluated the significance of SYT family members for GC patients, especially for prognosis. In the present study, we first used the online database to comprehensively explore the mRNA expressions and methylation levels of SYT family members and their significance in prognosis and underlying mechanisms in GC. We found that the expressions of SYT4, SYT9, and SYT14 at the mRNA and protein levels were significantly up-regulated in GC patients compared with the healthy controls, leading to the unsatisfactory OS and PFS for GC patients. Kanda M. et al. have shown that SYT8 is up-regulated in GC patients with peritoneal recurrence or metastasis, which is consistent with our results [18]. However, we further showed that SYT8 was not associated with OS for GC patients. Kanda M et al. have demonstrated that SYT7 is over-expressed in GC tissues, and it is significantly correlated with hepatic recurrence, metastasis, and poor prognosis [19]. Nevertheless, we found that the expression of SYT7 was similar between GC patients and healthy controls in this study. Such discrepancy could be attributed to the small sample size in above-mentioned study.

It has been reported that the methylation profile is crucial to evaluate the functional status of genes, since their expressions depend on the methylation status of the DNA CpG island [20,21]. Foschini MP et al. have shown that MAGEA family members are hypomethylated in male breast cancer (MBC), leading to their over-expression, which enhances the androgen receptor (AR) activity and AR therapy response [22]. Meanwhile, gene methylation has constituted an attractive research field in oncology, which is especially useful to predict the prognostic and therapeutic significance of cancer profile [23]. However, no study on methylation levels of SYT family members in GC has been published. In the present study, Pearson^’^s correlation analysis demonstrated that there was an inverse correlation between the mRNA expressions and methylation levels of SYT4, SYT9, and SYT14 in GC. Moreover, we further explored the association between the mRNA expressions and methylation levels of cg sites of SYT4, SYT9, and SYT14 in GC. The results showed that the methylation levels of two cg sites (cg12053284 and cg16222762) were negatively correlated with the expression of SYT4, the methylation levels of nine cg sites (cg02269161, cg03226737, cg08185661, cg14243481, cg16437728, cg18560328, cg22723056, cg24678137, and cg26945996) were inversely correlated with the expression of SYT9, and the methylation levels of six cg sites (cg02795029, cg07581146, cg15149095, cg19922137, cg25371503, and cg26158959) were negatively associated with the expression of SYT14. Subsequently, we found that the methylation levels of cg sites (cg12053284 and cg16222762) of SYT4 were not associated with OS and PFS for GC patients. These results indicated that the regulation of SYT4 promoter methylation was not the main route to regulate the expression of SYT4 in GC. Moreover, we demonstrated that low methylation levels of cg sites (cg02269161, cg03226737, cg08185661, cg16437728, cg22723056, and cg24678137) of SYT9 were associated with an unfavorable OS and PFS for GC patients. These results showed that these methylation cg sites might be potent prognostic indicators and therapeutic targets for GC. Furthermore, we demonstrated that the low methylation level of cg site (cg07581146) of SYT14 was correlated with an unsatisfactory OS and PFS for GC patients. This result indicated that the methylation cg site cg07581146 of SYT14 might also be a potent prognostic indicator and therapeutic target for GC. In addition, we found that the mRNA expressions of SYT4, SYT9, and SYT14 and their methylation levels were closely correlated with the T stage and pathological stage. Therefore, these results suggested that the expressions of SYT4, SYT9, and SYT14, and their methylation levels might play crucial roles in the development and progression of GC.

Several studies have pointed out that the tumor immune microenvironment plays a pivotal role in the development and prognosis of GC [24–27]. In the present study, we first showed that the expressions of SYT family members were correlated with the formation of the tumor immune microenvironment in GC. We found that the expression of SYT4 was positively correlated with the infiltration of CD4^+^T cells, CD8^+^T cells, DCs, macrophages, and neutrophils, whereas the expression of SYT9 had a positive association with the infiltration of B cells, CD4^+^T cells, CD8^+^T cells, DC, macrophages, and neutrophils. Indeed, the expression of SYT14 was positively associated with the infiltration of CD4^+^T cells and macrophages. These results demonstrated that SYT family members were mainly implicated in the immune microenvironment of GC through regulating these above-mentioned immune cells, which might be promising immunotherapy targets in the future.

Obviously, it was undeniable that there were some limitations in this study. First, all the data were obtained based on online databases, and further studies are required to confirm our findings. Second, it is necessary to verify the underlying mechanism between SYT family members and GC *in vivo* and *in vitro*.

### Conclusion

We demonstrated that SYT4, SYT9, and SYT14 were up-regulated, and their expressions were negatively correlated with the methylation levels in GC. Meanwhile, the over-expression of SYT4, SYT9, and SYT14 was significantly associated with an unsatisfactory OS and PFS. Furthermore, the expressions of SYT4, SYT9, and SYT14 played a pivotal role in immune cell infiltration. Therefore, SYT4, SYT9, and SYT14 might be potent prognostic indictors and promising immunotherapeutic targets for GC patients.

## Supplementary Material

Supplemental Material

## Data Availability

The HTSeq FPKM data, methylation450 profile, and clinical data with GC were downloaded from the TCGA database via UCSC Xena (https://xena.ucsc.edu/). All data generated in this study are included in this published article.

## Highlights

1.The expressions of SYT4, SYT9 and SYT14 were up-regulated in gastric cancer (GC).

2. The over-expression of SYT4, SYT9 and SYT14 contributed to an unsatisfactory overall survival (OS) and progression-free survival (PFS) for GC patients.

3. The expressions of SYT4, SYT9 and SYT14 played a pivotal role in immune cells infiltration in GC.

4. SYT4, SYT9 and SYT14 might be potent prognostic indictors and promising immunotherapeutic targets for GC patients.
