# Supplementary material for: Comprehensive analysis on the expression profile and prognostic values of Synaptotagmins (SYTs) family members and their methylation levels in gastric cancer
Source: Bioengineered. 2021 Jul 7;12(1):3550–65. doi: 10.1080/21655979.2021.1951059 (PMC8806422; doi:10.1080/21655979.2021.1951059)
Supplement: Supplemental Material [file KBIE_A_1951059_SM8427.zip › Supplement materialsclean.docx]

**Comprehensive analysis on the expression profile and prognostic values of Synaptotagmins (SYTs) family members and their methylation levels in gastric cancer**

Running title: Prognostic values of SYT family members

Mei-feng Yang^2*^, Xing-xing Long^3*^, Hong-sai Hu^4*^, Yu-ling Bin^4^, Xuan-ming Chen^5#^, Ben-hua Wu^1#^, Quan-zhou Peng^3#^, Li-sheng Wang^1#^, Jun Yao^1#^, and De-feng Li^1#^


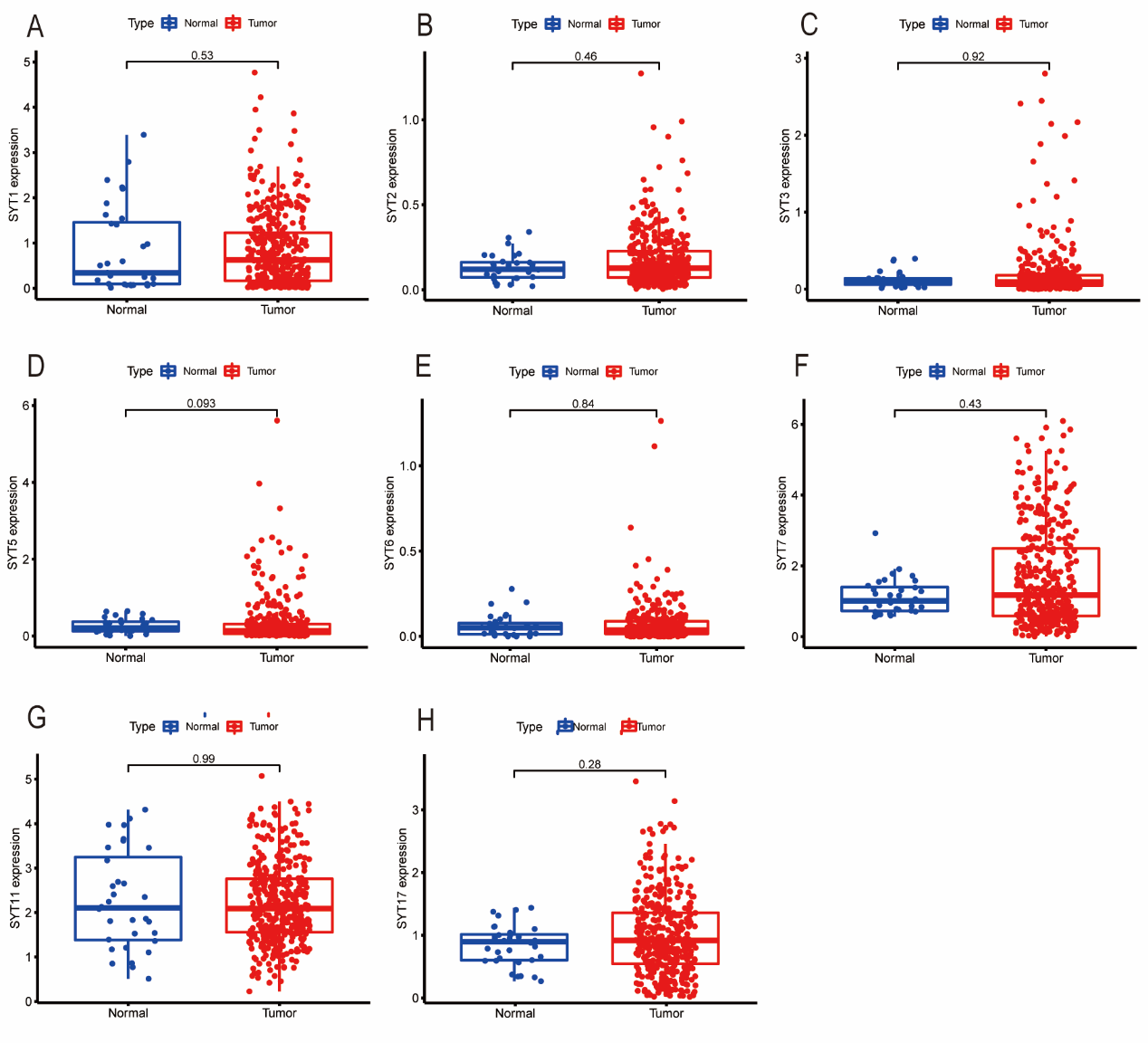


Figure S1: The SYTs family members mRNA expression in patients with GC compared to normal control from TCGA database. SYT1, SYT2, SYT3, SYT5, SYT6, SYT7, SYT11 and SYT17 had no significant difference compared gastric cancer to normal controls.


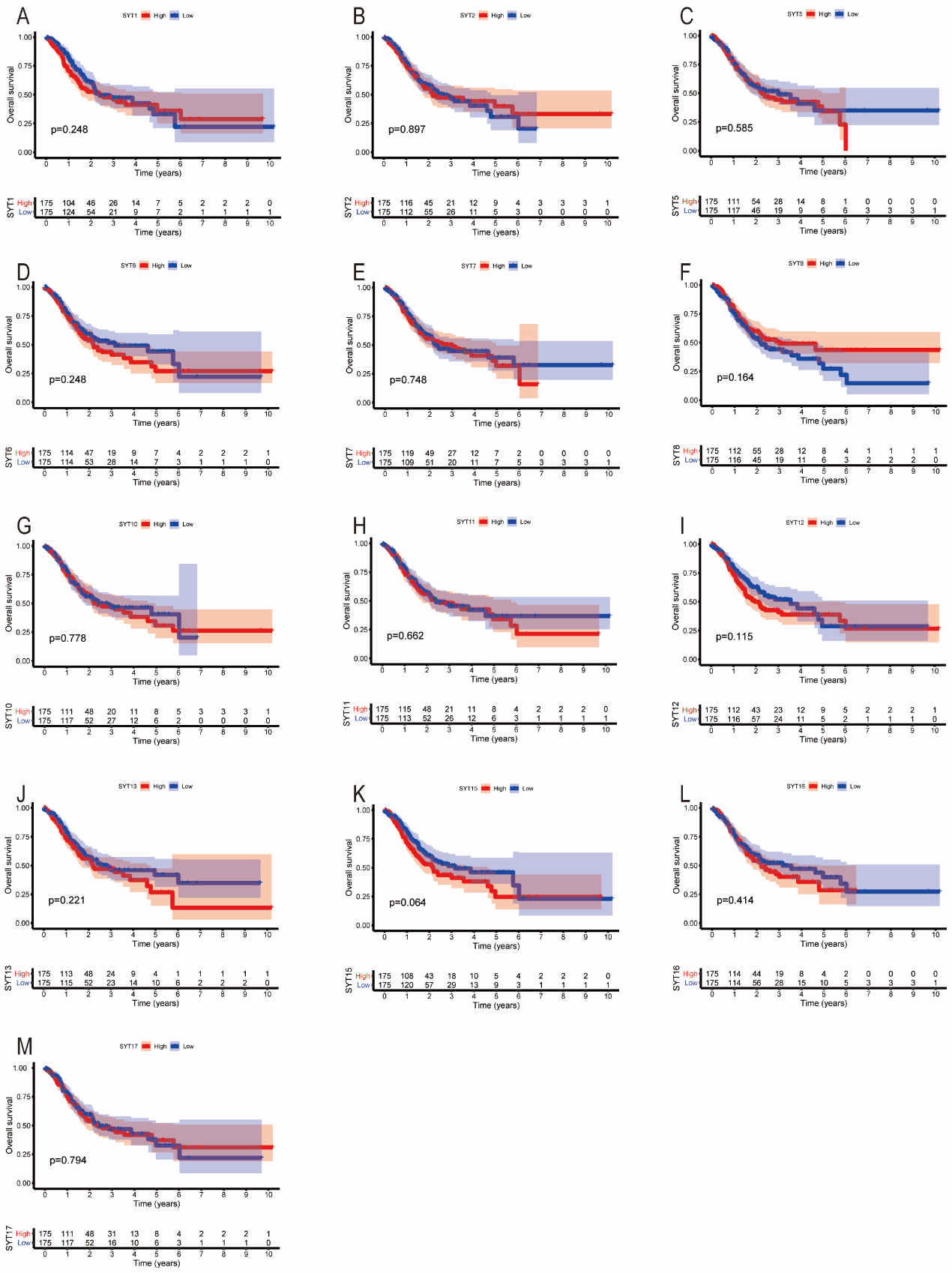


Figure S2: Overall survival (OS) value of SYTs members family mRNA expression in patients with GC. The expression of SYT1, SYT2, SYT5, SYT6, SYT7, SYT8, SYT10, SYT11, SYT12, SYT13, SYT15, SYT16 and SYT17 was not associated with OS.


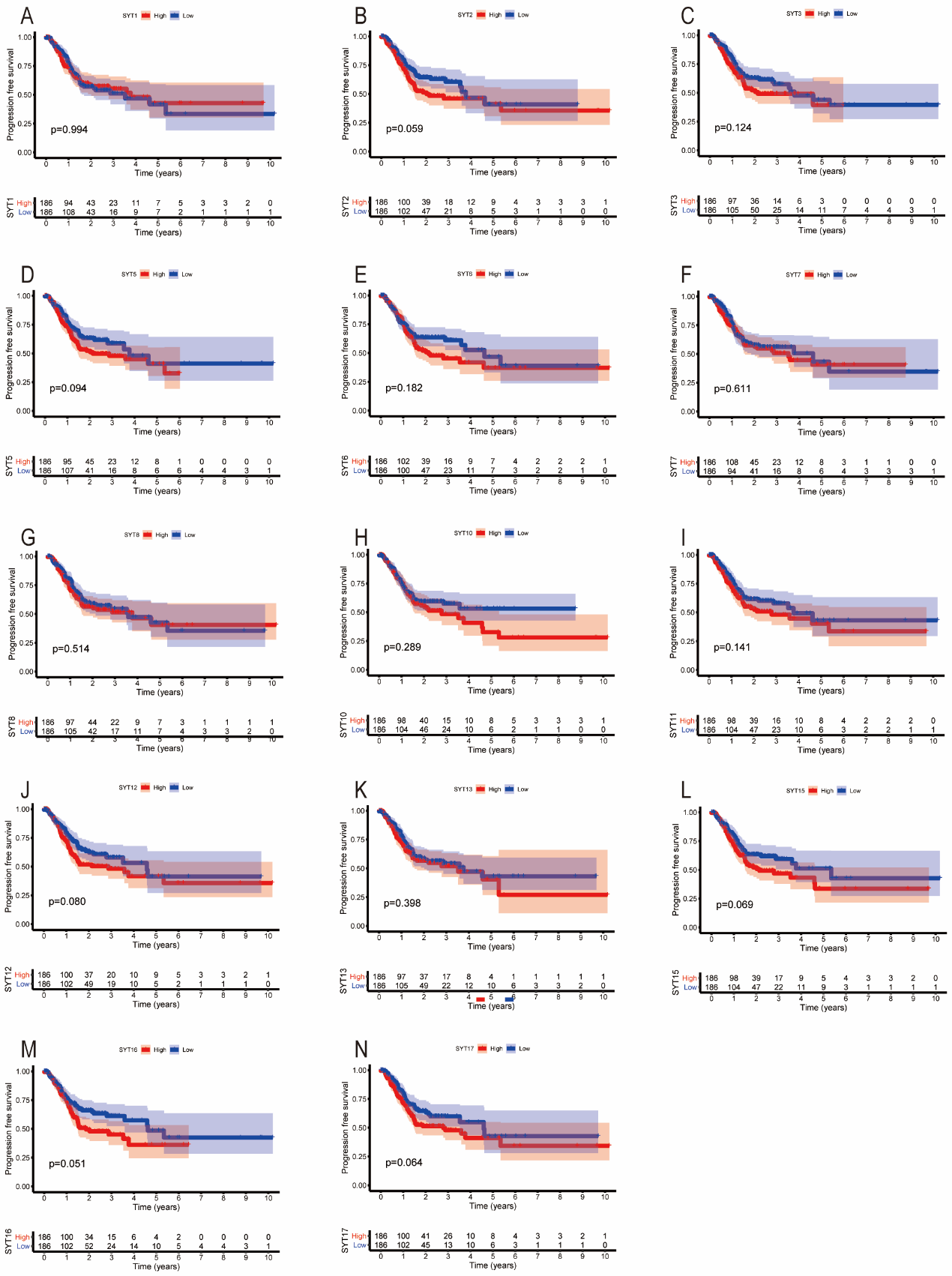


Figure S3: Progression free survival (PFS) value of SYTs members family mRNA expression in patients with GC. The expression of SYT1, SYT2, SYT3, SYT5, SYT6, SYT7, SYT8, SYT10, SYT11, SYT12, SYT13, SYT15, SYT16 and SYT17 was not associated with PFS.


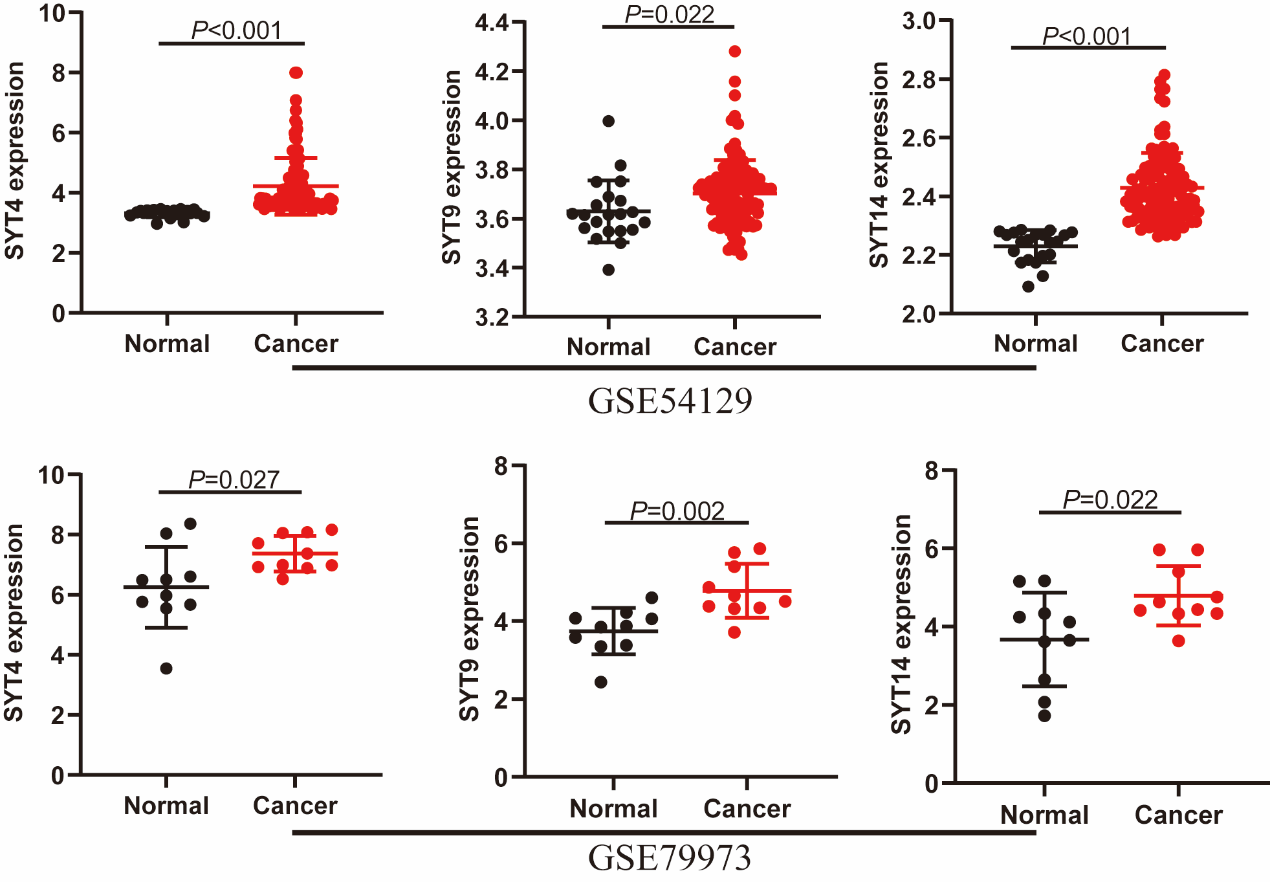


Figure S4: The SYTs family members mRNA expression in patients with GC compared to normal control from TCGA database. SYT4, SYT9 and SYT14 was up-regulated in the GSE54129 and GSE79973.


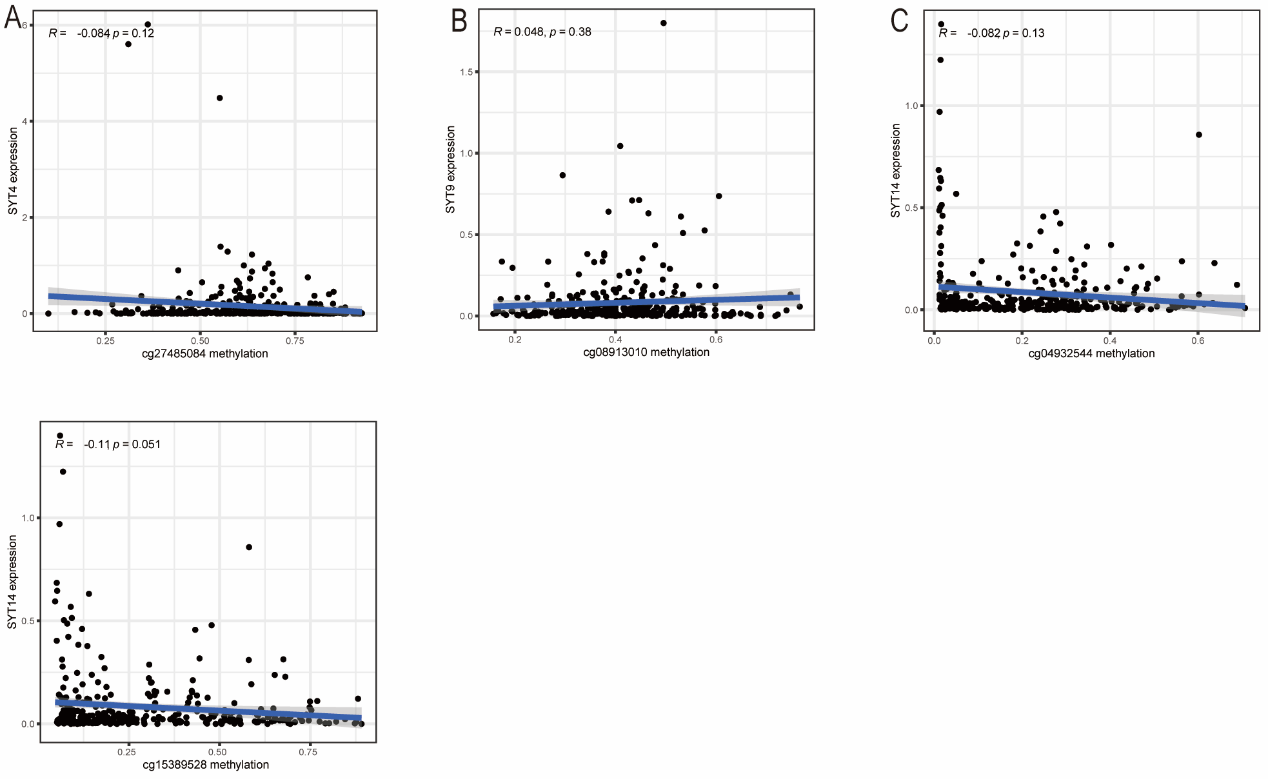


Figure S5: Correlation of SYTs family members expression and their methylation cg sites in GC. The cg27485084, cg08913010, cg04932544 and cg15389528 methylation cg sites was not correlated with the expression of SYTs family.


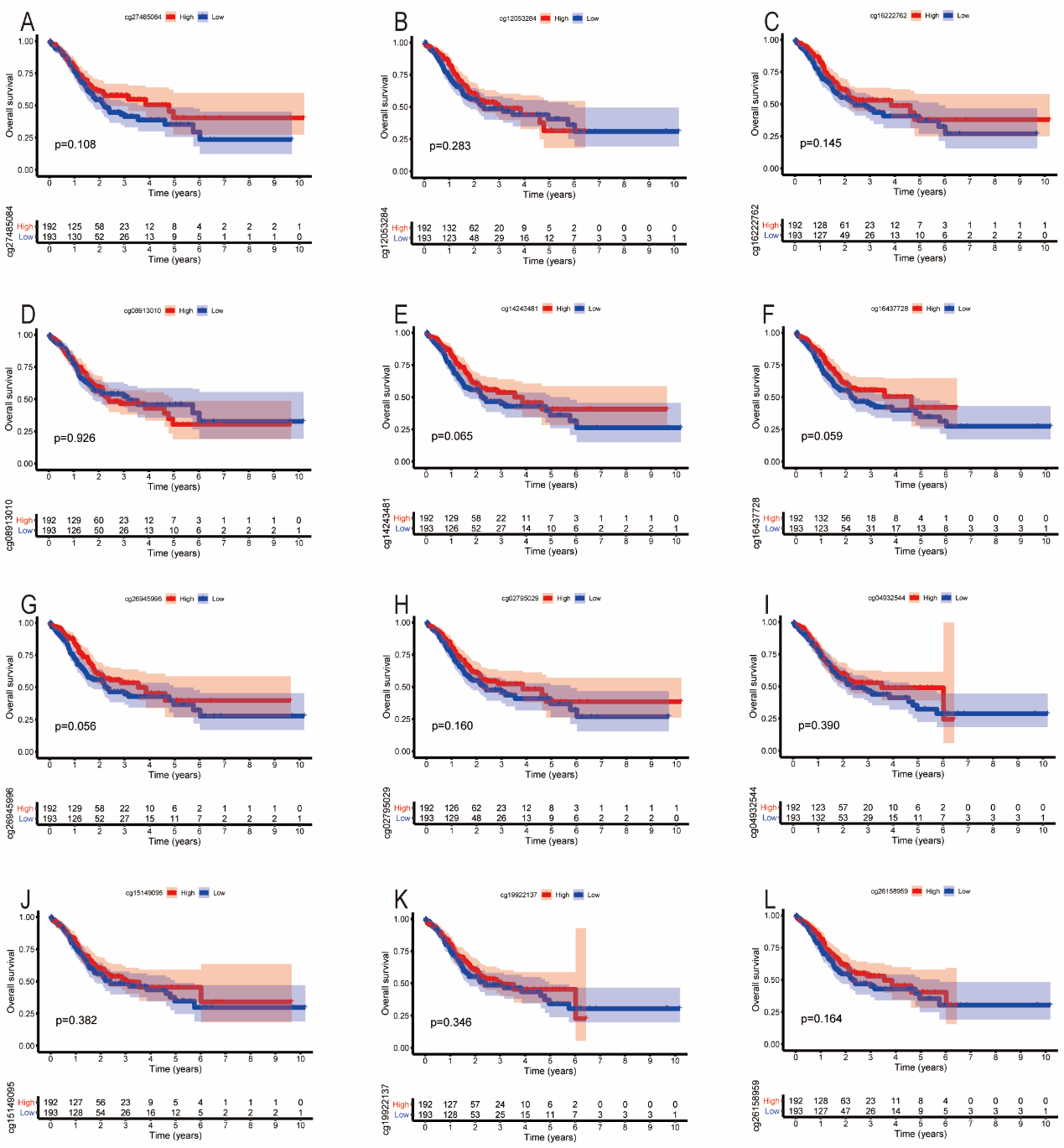


Figure S6: OS value of SYTs members methylation cg sites in patients with GC. The low methylation cg sites levels (cg27485084, cg12053284, cg16222762, cg08913010, cg14243481, cg16437728, cg26945996, g02795029, cg04932544, cg15149095, cg19922137 and cg26158959) were not associated with OS.


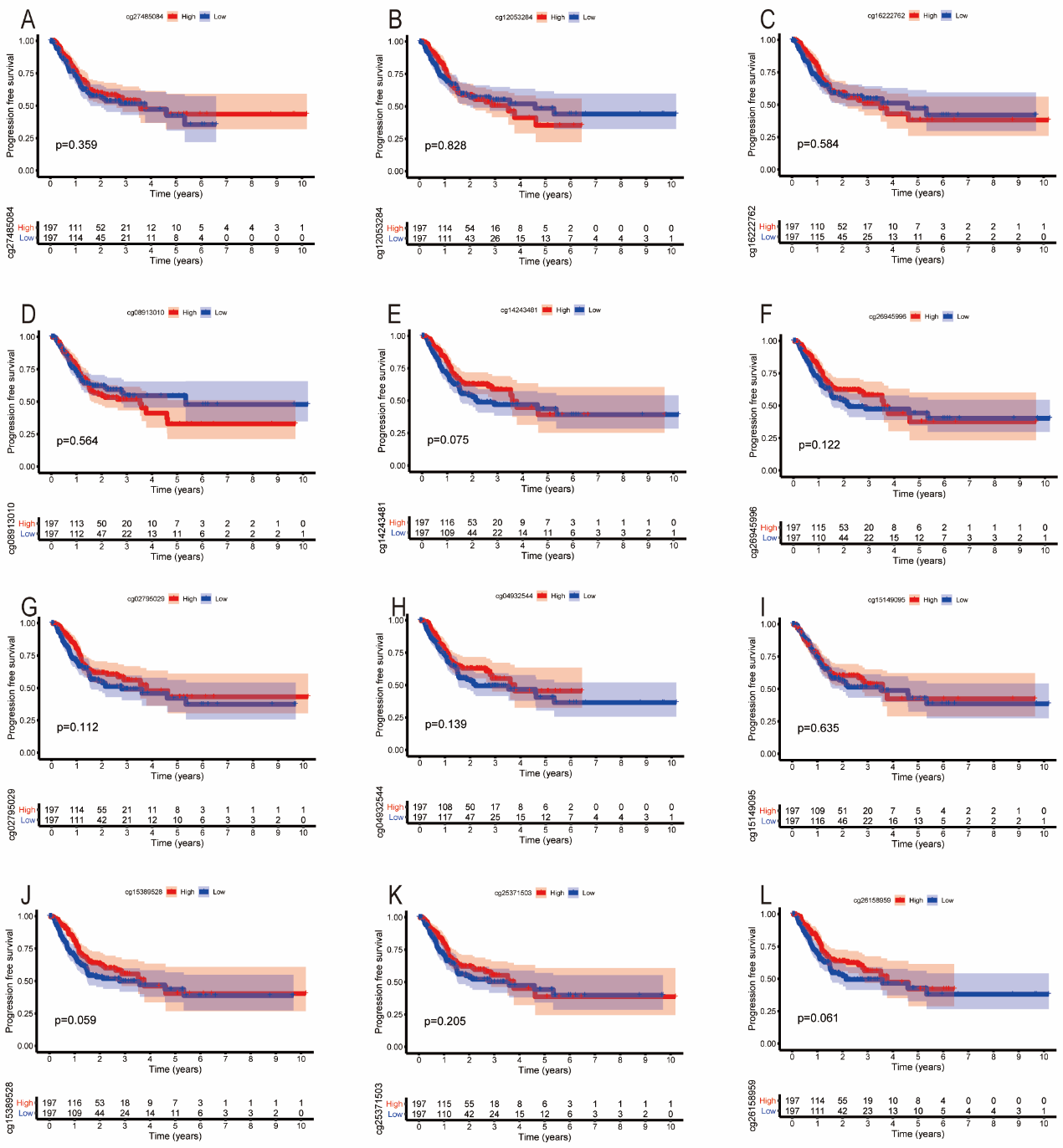


Figure S7: PFS value of SYTs members methylation cg sites in patients with GC. The low methylation cg sites levels (cg27485084, cg12053284, cg16222762, (cg08913010, cg14243481, cg26945996, cg02795029, cg04932544, cg15149095, cg15389528, cg25371503 and cg26158959) were not associated with PFS.
